# Paget Schroetter Syndrome: A Case Report On An Effort Induced Thrombosis

**DOI:** 10.51894/001c.144479

**Published:** 2025-10-01

**Authors:** Monika Kadari

**Affiliations:** 1 Department of Internal Medicine Garden City Hospital

## 89

### INTRODUCTION

Paget Schroetter Syndrome, which is the external compressive effect of costoclavicular junction enabling thrombus formation in the subclavian vein, is often referred to as effort induced thrombosis, with predominance in young healthy age, often athletic adults who indulge in vigorous upper extremity weightlifting. In our case, we can further do workup on ruling out anatomical causes of upper limb thrombosis.

### CASE REPORT

A 42-year-old African American male with no significant medical history presented to the Emergency Department with persistent pain and sudden swelling in his right upper limb for two days, which he attributed to his gym sessions. He reported no added symptoms or history of trauma, intravenous drug use, or clotting disorders.

His vital signs were stable, and physical examination revealed localized edema in the mid-forearm without tenderness. A duplex ultrasound confirmed a thrombus in the right axillary, brachial, and basilic veins, while a CT pulmonary embolism scan showed no embolism. Lower extremity Doppler ultrasound was negative for thrombus.

Laboratory tests, including CBC and metabolic panels, were normal, but lipid profile showed elevated triglycerides and LDL. D-dimer levels were high at 1149 ng/ml. Electrocardiogram showed normal sinus rhythm with lateral T wave inversions, and echocardiogram showed preserved ventricular function.

Mechanical thrombectomy was performed using the Inari ClotTriever device, with the thrombus being in an acute subacute phase. Balloon angioplasty addressed thoracic outlet stenosis due to Paget-Schroetter syndrome. Upon discharge, the patient was prescribed Eliquis (10 mg for seven days, then 5 mg daily for three months) and referred for further subclavian vein surgical decompression.

### DISCUSSION/CONCLUSION

In conclusion, the strategy of early CDT (catheter-dilated thrombolysis) and decompression of subclavian vein via first rib resection (FRR) is backed up by multiple studies, one of them being Hoexum et al. proving significant improvement in symptoms and recovery with reduced recurrence. Also, early surgical intervention shows better outcomes compared to conservative management, especially if done in the first month as per study done by Urschel and Razzuk.

